# Is Day-4 morula biopsy a feasible alternative for preimplantation genetic testing?

**DOI:** 10.1371/journal.pone.0238599

**Published:** 2020-09-11

**Authors:** Raoul Orvieto, Baruch Feldman, Marine Wiesel, Hagit Shani, Adva Aizer

**Affiliations:** 1 Department of Obstetrics and Gynecology, Sheba Medical Center, Ramat-Gan, Israel; 2 Sackler School of Medicine, Tel Aviv University, Tel Aviv, Israel; 3 The Tarnesby-Tarnowski Chair for Family Planning and Fertility Regulation, Sackler Faculty of Medicine, Tel-Aviv University, Tel Aviv, Israel; 4 Danek Gertner Institute of Human Genetics, Sheba Medical Center, Ramat-Gan, Israel; IRCCS San Raffaele Scientific Institute, ITALY

## Abstract

**Objective:**

To assess the efficacy and clinical outcome of PGT-M undertaken on Day-3, Day-4 and Day-4 “delayed” embryos that were unsuitable for biopsy on Day-3.

**Design and setting:**

Cohort-historical study of all consecutive patients admitted to the IVF-PGT-M program in a large tertiary center.

**Main outcome measure(s):**

The pregnancy rates and the percentages of complete, incomplete diagnosis, PCR failure, abnormal embryos in PGT of Day-3 cleavage-stage, Day-4 and Day-4 “delayed” embryos.

**Patients and methods:**

We reviewed the medical files of all consecutive patients admitted to our IVF for a fresh IVF-PGT-M cycle. Patients were divided into 3 groups according to the day of blastomere biopsy: Day 3 cleavage-stage, Day-4 morula and Day-4 “delayed” embryos. The laboratory data, genetic diagnostic and clinical results were collected and compared between the different study groups.

**Results:**

Nine hundred and six patients underwent PGT-M cycles in our PGT program: 747, 127 and 32 in the Day-3, Day- 4 and Day-4 “delayed” groups, respectively. Ongoing pregnancy rates per transfer and per patient (15.8% and 9.4%, respectively) were non-significantly lower in the Day-4 “delayed”, compared to Day-3 (21.4% and 17.5%, respectively) and Day-4 (24.3% and 19.7%, respectively). When comparing ALL morulas (Day-4 and Day-4 “delayed”) to ALL cleavage-stage embryos (Day-3, Day-4 and Day-4 “delayed”), a significantly higher ongoing pregnancy rate was demonstrated following the transfer of embryos derived from morula biopsy, as compared to biopsy at the cleavage-stage (33.3% vs 20.5%, p<0.03, respectively).

**Conclusion:**

Day-4 embryo biopsy is feasible and yields comparable and even higher ongoing pregnancy rate if undertaken at the morula stage. Further studies evaluating the cumulative live-birth rate per started cycles in Day-3 vs Day-4 embryo biopsy for PGT-M are warranted.

## Introduction

Preimplantation genetic testing (PGT) allows couples, at risk for transmitting a serious genetic disease, to select unaffected embryos for transfer, and enable the birth of healthy offspring. The main indications for PGT are specific monogenic aberrations and gender related disorders (PGT-M), structural and numerical chromosomal imbalances (PGT-SR), as well as aneuploidy screening (PGT-Aneuploidy PGT-A) [1, 2]. Based on single embryonic cell testing, PGT-M has already been applied in a wide range of structural and numerical chromosomal imbalances, monogenic disease, HLA typing, etc., with PGT- polymerase chain reaction (PCR), the method of choice for amplifying the small DNA content achieved from the blastomere biopsy.

Based on a 2013 study [3], demonstrating that cleavage-stage biopsy markedly reduced embryonic reproductive potential compared to trophectoderm biopsy, during the last half decade, a shift toward blastocyst rather than cleavage-stage biopsies appeared, mostly in PGT-Aneuploidy setups. A closer look at this study reveals that their “misleading conclusion” results, not from the detrimental effect of cleavage-stage embryos biopsy that showed an acceptable 30% sustained implantation rate, but due to their “incredibly” high (50%) sustained implantation of the un-biopsied cleavage-stage embryos, which was comparable/equivalent to the sustained implantation rates of the biopsied and un-biopsied blastocysts. (51% vs. 54%, respectively). The latter contradicts the common knowledge, as present in a recent Cochrane review [4], that clinical pregnancy/live birth rates following fresh transfer are higher in blastocyst compared to cleavage-stage transfers. Moreover, if 36% of women achieve clinical pregnancy after fresh cleavage stage transfer (figure similar to that achieved following the transfer of biopsied cleavage stage embryos in 2013 study), between 39% and 46% would do so after fresh blastocyst stage transfer [4].

Not surprisingly, according to the ESHRE PGD Consortium data collection XIV-XV [5] on PGT-M, day 3 cleavage-stage embryo biopsy was still the most frequently used (93% of cycles), while the use of blastocyst biopsy remained low (2%). Moreover, PCR was the most widely used first-line method of DNA amplification (93% of cycles). Results that were very similar to the previous data collection [6].

In our PGT-M program, we use blastomere biopsies from 3 different developmental-stage embryos: (a) High quality (>6 blastomere and <15% fragmentation) Day-3 cleavage-stage embryos. (b) Whenever Day-3 coincident with weekend/holiday (non-working day), the embryos are cultured for one more day and undergo biopsy on Day-4- at the morula stage; and (c) in case where no high quality embryos were suitable/available for biopsy on Day 3, embryos are cultured for one more day, and if caught-up, they undergo Day-4 blastomere biopsy.

Prompted by the aforementioned observations we sought to assess the efficacy and clinical outcomes of PGT-M undertaken on Day-3/4 cleavage-stage and Day-4 morula stage embryos.

## Patients and methods

### Patients

We reviewed the computerized files of all consecutive patients admitted to our IVF-PGT-M program from January 2006 to February 2020. We included only patients undergoing PGT-M, based on multiplex PCR programs designed for haplotyping using informative microsatellites markers [7] that reached the ovum pick-up (OPU) stage and had at least one embryo available for genetic evaluation. All data were fully anonymized before we accessed them. The study was approved by the IRB of the Sheba Medical Center ethical committee (IRB approval#: 9918).

All the usual indications for IVF/ICSI and accepted protocols for ovarian stimulations (described in [7] & [8]) were included. Laboratory procedures and molecular diagnosis were thoroughly presented elsewhere [7]. Progesterone luteal support was started with either daily 600 mg micronized progesterone soft gel vaginal capsules (Utrogestan, Besins, Iscovesco, C.T.S., Petach Tikva, Israel) in three divided doses, or vaginal progesterone 90 mg (Crinone; Merck Serono, Hellerup, Denmark) once a day. Progesterone luteal support was initiated 1 day after oocyte pick-up. Ongoing pregnancy was defined when the pregnancy had completed ≥12 weeks of gestation.

### PGT

Day-3 embryos underwent blastomere biopsy as previously described [7] (Day-3 group). In case where no high quality embryos were suitable/available on Day-3 for biopsy (>6 blastomere and <15% fragmentation), embryos were cultured for one more day, and if caught-up, they underwent Day-4 blastomere biopsy (Day-4 “delayed” group). Moreover, whenever Day-3 coincident with weekend/holiday (day off), the embryos were cultured for one more day and underwent biopsy of Day-4, at the morula stage. i.e. embryo composed of 12–32 blastomeres and all of the blastomeres are in the compaction process (Day-4 group). Day-4 embryos were placed in calcium magnesium free media (SAGE, CooperSurgical) for several minutes until they were de-compacted. Thereafter, the biopsy technique was performed as previously described for day-3 embryo biopsy [7].

Molecular diagnoses of each embryo within the 3 study groups are classified as previously described [7]:" **Complete diagnosis**–unaffected or affected embryo according to the genetic disorder examined; **Incomplete diagnosis**—suspected allele dropout or recombination; **PCR failure**–no DNA is available for diagnosis; **Abnormal**–the embryo has abnormal assembly of alleles–i.e. any structure different from one maternal and one paternal alleles matching the known haplotype, e.g. trisomy, monosomy or uniparental disomy".

### Statistics

Differences in variables between the study groups were statistically analyzed with chi-square test as appropriate. A p-value of <0.05 was considered significant.

## Results

Nine hundred and six patients underwent PGT-M cycles in our PGT program between January 2006 and February 2020: 747, 127 and 32 in the Day-3, Day- 4 and Day-4 “delayed” groups, respectively.

The laboratory/ embryological and genetic data are presented in Table 1. While no significant in-between group differences were observed in fertilization rates, the percentage of embryos available for biopsy was higher in Day-4 compared to Day-3 groups. Moreover, Day-4 “delayed” group had the lowest embryo transfer rate (59.4%), compared to Day-3 (81.8%, p<0.01) and Day-4 (81.1%, p<0.02) groups. Ongoing pregnancy rates per transfer and per patient (15.8% and 9.4%, respectively) were non-significantly lower in the Day-4 “delayed”, compared to Day-3 (21.4% and 17.5%, respectively) and Day-4 (24.3% and 19.7%, respectively). Same trend was observed also when evaluating the implantation rates.

**Table 1:** The clinical, embryological and genetic outcomes in the different study groups.

	Day-3 group	Day-4 group	Day-4 “delayed” group	p-value
Patients’ age				
(median, yrs)	32	33	30	NS
# of patients/cycles	747	127	32	
Indications for PGT				
Single gene disorders	692	121	28	
Chromosomal abnormalities	35	2	4	
Sex selection	20	4	0	
# oocytes retrieved	7716	1250	238	
# fertilized oocytes (%)	5369 (69.6%)	871(69.7%)	158(66.4%)	NS
# embryos Bx	4513	770	135	
(% of fertilized oocytes)	(84.1%)^	(88.4%)^	-85.40%	^p<0.001
# of embryo transfers (% per patient)	611(81.8%)^	103 (81.1%)*	19 (59.4%)*^	^p<0.01;*p<0.02
# of gestational sacs (implantation rate %)	172 (18.1%)	32 (19.9%)	4 (15.4%)	NS
# Ongoing pregnancy	131	25	3	
(% per transfer)	(21.4%)^	(24.3%)*	(15.8%)*^	^p = 0.12; *p = 0.098
(% per patient)	-17.50%	-19.70%	-9.40%	NS
**Tested embryos**				
Complete (%)	3253(72.1%)	542(70.4%)	96(71.1%)	NS
*Unaffected*	1677(37.2%)^	271 (35.2%)*	35 (25.5%)*^	^p<0.005; *p<0.03
Incomplete (%)	277(6.1%)	53(6.9%)	8(5.9%)	NS
PCR Failure (%)	524(11.6%)	96(12.4%)	20(14.8%)	NS
Abnormal (%)	459(10.2%)	79(10.2%)	11(8.1%)	NS

Complete—affected or unaffected; Incomplete–suspected ADO or recombination; PCR failure–no embryonal DNA is available for PCR; Abnormal–the embryo has abnormal assembly of alleles–i.e. any configuration of alleles different from one maternal and one paternal alleles; Unaffected- healthy transferable embryos.

The molecular diagnoses are presented in Table 1. While no significant differences were observed in the percentages of complete, incomplete diagnosis, PCR failure or abnormal embryos, between the study groups, the Day-4 “delayed” yielded a significantly lower unaffected embryos compared to the Day- 3 (25.5% vs 37.2%, p<0.005, respectively) and Day-4 groups (25.5% vs 35.2%, p<0.03, respectively).

Table 2 presents the ongoing pregnancy rate according to the embryos developmental stage in the different study groups. Day-4 morula achieved significantly higher ongoing pregnancy rates compared to cleavage-stage embryos at Day-3, Day-4 and Day-4 “delayed” (Table 2A). Moreover, when comparing ALL morulas (Day-4 and Day-4 “delayed”) to ALL cleavage-stage embryos (Day-3, Day-4 and Day-4 “delayed”), a significantly higher ongoing pregnancy rate was demonstrated following the transfer of morula, as compared to cleavage-stage embryos (33.3% vs 20.5%, p<0.03, respectively. Table 2B).

**Table 2:** Ongoing pregnancy rate according to the embryos developmental stage in the different study groups.

**[A]**							
	Day-3 group	Day-4 group			Day-4 “delayed” group		
# of patients/cycles	641/747	127/127			32/32		
Embryos developmental	Cleavage-stage	Cleavage-stage	Morula	Total	Cleavage-stage	Morula	Total
# of embryos	4513	458	312	770	100	35	135 (% of total)
# of embryo transfers	611	42	61	103	11	8	19
# Ongoing pregnancy	131	5	20	25	0	3	3
(% per transfer)	(21.4%)^	+(11.9%)	^+*(32.8%)	(24.3%)	*~0	~(37.5%)	(15.8%)
(% per patient)	^ (20.4%)	+(11.9%)	^+*(32.8%)	(24.3%)	*~0	~(37.5%)	(15.8%)
**[B]**							
		Number of embryo transferred		Ongoing pregnancy (% per transfer)			
Cleavage-stage	Day-3	611		131 (21.4%)			
embryos	Day4	42		5 (11.9%)			
	Day-4 “delayed	11		0			
	TOTAL	664		136 (20.5%)*			
Morula	Day-3	0					
	Day4	61		20 (32.8%)			
	Day-4 “delayed	8		3 (37.5%)			
	TOTAL	69		23 (33.3%)*			

^p = 0.06

*~p = 0.001

+p<0.01

*P<0.03

## Discussion

In the present study of patients undergoing fresh IVF treatment cycle, utilizing PGT-M based on multiplex PCR programs, ongoing pregnancy rates per transfer and per patient were comparable between Day-3 (21.4% and 17.5%, respectively) and Day-4 (24.3% and 19.7%, respectively) embryos biopsy. Moreover, if biopsy should be delayed for technical reasons, Day-4 embryo biopsy at the morula stage is feasible and yield a significantly higher ongoing pregnancy rate, compared to cleavage-stage embryo biopsy. Day-3 embryos not suitable for biopsy should be cultured to Day-4. If the embryos will catch-up to the morula stage, embryo biopsy for PGT-M is recommended. However, embryo biopsy should be avoided from Day-4 embryos that did not develop beyond the cleavage-stage.

Our overall performance, and the outcome of PGT-M cycles were comparable to the ESHRE consortium data collection XIV–XV [5]. The fertilization rates (61.8%) and percentages of embryos undergoing biopsy per fertilized oocyte (74.3%,), were comparable to our experience in Day-3 biopsy (69.6% and 84.1%, respectively). Of the embryos undergoing biopsy, overall, 72.1% were designated to have a complete diagnosis–unaffected or affected embryo according to the genetic disorder examined. Moreover, of the biopsied embryos, the percentages of unaffected/transferable embryos were 37.2% overall, figure that is in line with the ESHRE data [5], reporting that 40.9% of diagnosed embryos were genetically transferable. Moreover, a closer look at the data presented by De Rycke [5] on PGT-M cycles performed for single gene disorders using PCR, revealed delivery rates per OPU and per embryo transfer of 20% and 26%, respectively, which are in accordance with our figures. Of notice, in our general IVF population, between 2016 and 2019, we conducted 2263 OPU, with 31% and 28.1% ongoing pregnancy rate per embryo transfers, in patients age <35 yrs and <40 yrs, respectively.

A recent study by Theobald et al. [9] presented the status of PGT in the UK and USA between 2014 to 2016. According to Theobald et al., in 2016, patients undergoing PGT-M/SR yielded 18.9% and 29.8% live birth rates per OPU/transfer, respectively. While these figures are higher than those reported by us or De Rycke [5], it should be emphasized that Theobald et al. have reported on the overall PGT outcomes, including blastocyst transfer. Moreover, their data related to the years 2014 to 2016, while we reported on our experience between 2006 to 2020.

Our observations report on similar results when performing PGT-M on Day-4 embryos, compared to Day-3. However, as expected, more developed embryos at the morula stage (Day 4) yield better pregnancy rate per transfer compared to cleavage-stage (Day 3) embryos (Table 2A and 2B). Moreover, whenever embryos are not suitable for biopsy on Day-3, if they will catch-up to the morula-stage on Day-4, their reproductive potential is practically restored and they should be considered for PGT-M. On the other hand, those that did not catch-up to the morula stage on Day-4, and remained at the cleavage-stage, yielded ZERO pregnancy rate, and therefore their biopsy should be avoided.

These observations are in line with the observed higher pregnancy rate per transfer, while comparing Day-5 blastocyst to Day-3 cleavage-stage embryo transfers [4]. The more developed the embryo transferred the higher the pregnancy rate. However, it should be clarified that extended culture may harm embryonic development due to suboptimal culture conditions [10], as already demonstrated by Racowsky et al. [11] and Xiao et al. [12], who reported an improved pregnancy rate when transferring cleavage stage embryos versus blastocyst, when only a single embryo was available.

## Conclusion

It might be therefore concluded, that in PGT-M cycles, Day-4 embryo biopsy is feasible and yields comparable and even higher ongoing pregnancy rate if undertaken at the morula stage. Further studies evaluating the cumulative live-birth rate per started cycles in Day-3 vs Day-4 embryo biopsy for PGT-M are warranted.
